# Cep169, a Novel Microtubule Plus-End-Tracking Centrosomal Protein, Binds to CDK5RAP2 and Regulates Microtubule Stability

**DOI:** 10.1371/journal.pone.0140968

**Published:** 2015-10-20

**Authors:** Yusuke Mori, Yoko Inoue, Sayori Tanaka, Satoka Doda, Shota Yamanaka, Hiroki Fukuchi, Yasuhiko Terada

**Affiliations:** Department of Chemistry and Biochemistry, School of Advanced Science and Engineering, Waseda University, 3-4-1 Ohkubo, Tokyo 169-8555, Japan; Cancer Research UK London Research Institute, UNITED KINGDOM

## Abstract

The centrosomal protein, CDK5RAP2, is a microcephaly protein that regulates centrosomal maturation by recruitment of a γ-tubulin ring complex (γ-TuRC) onto centrosomes. In this report, we identified a novel human centrosomal protein, Cep169, as a binding partner of CDK5RAP2, a member of microtubule plus-end-tracking proteins (+TIPs). Cep169 interacts directly with CDK5RAP2 through CM1, an evolutionarily conserved domain, and colocalizes at the pericentriolar matrix (PCM) around centrioles with CDK5RAP2. In addition, Cep169 interacts with EB1 through SxIP-motif responsible for EB1 binding, and colocalizes with CDK5RAP2 at the microtubule plus-end. EB1-binding–deficient Cep169 abolishes EB1 interaction and microtubule plus-end attachment, indicating Cep169 as a novel member of +TIPs. We further show that ectopic expression of either Cep169 or CDK5RAP2 induces microtubule bundling and acetylation in U2OS cells, and depletion of Cep169 induces microtubule depolymerization in HeLa cells, although Cep169 is not required for assembly of γ-tubulin onto centrosome by CDK5RAP2. These results show that Cep169 targets microtubule tips and regulates stability of microtubules with CDK5RAP2.

## Introduction

The centrosome consists of a pair centrioles surrounded by the pericentriolar matrix (PCM). In animal cells, microtubules (MTs) are organized from the centrosome/microtubule-organizing center (MTOC) [1]. MTs are nucleated by γ-tubulin ring complex (γ-TuRC) that is targeted to the MTOC [2]. *Drosophila melanogaster* centrosomin (CNN) is required for centrosomal maturation and recruitment of γ-TuRC onto centrosomes through its amino-terminal CNN Motif 1 (CM1) in CNN [3], which is highly conserved in mammalian CDK5RAP2 (also known as Cep215), S. pombe Mto1p and Pcp1p [4, 5]. CDK5RAP2 is a functional human homolog of CNN required for a centrosomal maturation and planer spindle orientation in neuroblast, and is found to be mutated in primary microcephaly (MCPH), a neuro developmental disorder characterized by reduced brain size [6]. However, molecular mechanisms of CDK5RAP2 are poorly understood.

The ends of growing MTs accumulate a set of diverse factors known as MT plus-end-tracking proteins (+TIPs), which control MT dynamics and organization. +TIPs are specialized MAPs that are conserved in all eukaryotes and accumulates at growing MT plus-ends. These proteins include end-binding protein 1 (EB1), a prototypic member of +TIPs, adenomatous polyposis coli (APC) [7–9], cytoplasmic linker proteins (CLIPs), and CLIP-associating proteins (CLASPs) [10–12]. +TIPs form a complex interaction network to achieve their localization to growing MT plus-ends. Among them, EB1 forms the core machinery for MT tip tracking and targets additional +TIPs to MT ends [13–15]. Indeed, CDK5RAP2 interacts directly with EB1 through a short hydrophobic (S/T)x(I/L)P sequence motif (SxIP), sequence region enriched in serine and proline residues responsible for EB1 interaction, at MT plus-ends to regulate MT dynamics [16].

In this study, we identified a novel MT plus-end-tracking and centrosomal protein, Cep169, as a binding partner of CDK5RAP2. We show that Cep169 and CDK5RAP2 coloclalize at both centrosomes and MT plus-ends. Cep169 contains the three SxIP motifs that have been found in several +TIPs. Although Cep169 is not required for recruitment of γ-tubulin onto centrosomes, Cep169 promotes MT assembly. These results show that Cep169 forms a plus-end complex with EB1 and regulates MT stability with CDK5RAP2.

## Materials and Methods

### Cell culture and RNA interference experiments

HeLa, U2OS (ATCC) and 293T were cultured at 37°C in 5% CO_2_ in DMEM containing 10% FBS. pcDNA5/FRT/TO-FLAG Cep169 was transfected into Flip-In^TM^T-REx^TM^-HeLa cells (Invitrogen) to establish an inducible FLAG-tagged Cep169 stable cell line. For RNA interference, cells were plated 24 h before transfection with Oligofectamine or Lipofectamine RNAi MAX (Invitrogen) performed according to the manufacture’s instructions. The following oligonucleotides were used: control si*Luc* (5′-CGUACGCGGAAUACUUCGATT-3′) and si*Cep169* (5′-CUUGCCCAGGCCAACGAAAATT-3′) or (5′-CCUCAUCUCAGGUGAAAAGTT-3′). Oligonucleotides were obtained from Japan Bioservice. For lentivirus production of shRNA against human *CDK5RAP2*, pHR-CMVΔR8.2Δvpr and pHR-CMV-VSV-G were cotransfected with either pLKO.1-puro-sh-CDK5RAP2 to generate shRNA virus or pHR-hCMV*-1-SV-puro-Luciferase for control shRNA. HeLa and U2OS cells infected with the indicated viruses were selected with 3 μg/ml of puromycin. Lentiviral production and cell transfection were performed using standard protocols [17].

### Yeast-two hybrid screening

CM1 domain of human CDK5RAP2 (58–196) [“(58–196)” indicates amino acids 58 to 196] was fused in-frame to the GAL4 DNA-binding domain using the pGBKT7 vector (CLONTECH). The resulting “bait” plasmid was used to screen a 9.5/10.5-day-old mouse embryo cDNA two-hybrid library (a gift from S. Hollenberg) in screens for CDK5RAP2-binding proteins by the yeast two-hybrid method as described previously [3].

### Plasmid constructions and transfection

The entire human cDNAs coding sequences of Cep169/NCKAP5L were cloned by PCR from HeLa cDNA libraries and ligated into expression vectors of pEGFP-C, pCMV TAG2 and pcDNA5/FRT/TO (Invitrogen), respectively. Human CDK5RAP2 gene (Kazusa DNA Res. Inst.) was subcloned into pEGFP-C1 and pCMV Tag2, respectively. To generate lentivirus-based RNA interference (RNAi) transfer plasmid pLKO.1-puro-sh-CDK5RAP2, the oligonucleotides (forward, 5′-CCGGCAGCAGAACTGCTATTTAATGCTCGAGCATTAAATAGCAGTTCTGCTGTTTTTG-3′, and reverse, 5′-AATTCAAAAACAGCAGAACTGCTATTTAATGCTCGAGCATTAAATAGCAGTTCTGCTG-3′) were annealed and then inserted into pLKO.1-puro vector (a gift from S. A. Stewart and P. A. Sharp).

siRNA-resistant FLAG-Cep169 wild type (WT) and SxAA123 were constructed by mutagenesis using PrimeSTAR Mutagenesis Kit (TaKaRa), using the following primer pair: 5′-CCAAGCTAATGAGAACCAGCGGGAGACTTAT-3′ and 5′-TTCTCATTAGCTTGGGCAAGTGCCGAGTTC-3′; 5′-ATCGCAAGTCAAGAGCAAGCTCCAAATTGGC-3′ and 5′-GCTCTTGACTTGCGATGAGGAATGGGGGTGG-3′. FLAG-Cep169 SxAA1, SxAA2 and SxAA3 were constructed by mutagenesis, using the following primer pair: 5′-TCGCGAGCCGCCTGTCGGAACAGTGGCT-3′ and 5′-GACAGGCGGCTCGCGAGTTCCGGGGGAG-3′; 5′-ACCAAGGCCGCTTCCAAGTCACCAACCA-3′ and 5′-TGGAAGCGGCCTTGGTGGGGCTGCTGTG-3′, 5′-AGCAAGGCGGCAGCGCTGAACCGCCGCA-3′ and 5′-GCGCTGCCGCCTTGCTCTTCTTAAGGCC-3′, respectively.

Purified plasmid DNA was mixed with BioT (Bioland Scientific) or Lipofectamine 2000 (Invitrogen), and added to HeLa, U2OS, or 293T cells.

### Antibody production and purification

Cep169 (1–100) [“(1–100)” indicates amino acids 1 to 100] was produced as glutathione S-transferase (GST) fusion protein in *E*. *coli* and purified using GST column (Amersham Biosciences) as described previously [16]. The purified protein was injected into rabbits to raise polyclonal anti-Cep169 antisera. To affinity purify the anti-Cep169 antibody, immunized sera were first depleted of anti-GST antibody using a GST column. The resulting sera were further purified using GST-Cep169 (1–100) immobilized with Affigel-10 (Bio-Rad Laboratories).

### Immunoprecipitations and pull-down experiments

293T cells were transfected with EGFP-Cep169 and FLAG-CDK5RAP2 and extracted by extraction buffer (20 mM Tris-HCl at pH 7.5, 100 mM NaCl, 5 mM MgCl_2_, 0.2% Nonidet P-40, 10% glycerol, 1 mM NaF, 1 mM Na_3_VO_4_, 20 mM β-glycerophosphate, 10 mM β-mercaptoethanol, 0.2 mM PMSF, Protease Inhibitor Cocktail [Nacarai Tesque]) by two rounds of freezing and thawing as described previously [16]. An equal amount of each lysates were incubated with anti-FLAG antibody or rabbit IgG coupled to protein G beads (Amersham) for 1 h at 4°C. After washing the beads three times with extraction buffer, the immune complexes were analyzed by Immunoblotting. For *in vitro* GST pull-down assay, the recombinant protein of GST-EB1 was expressed in *Escherichia coli* and purified by affinity chromatography on glutathione-sepharose 4B (GE Healthcare). The recombinant protein of GST-EB1 and glutathione sepharose-beads were mixed with cell extracts from 293T transiently expressed with FLAG-Cep169 wild type or SxAA mutant. Consequent to incubation for 12 h at 4°C under continuous rotation beads were washed in lysis buffer three times. Proteins were eluted in Laemmli buffer, separated by SDS-PAGE and analyzed with immunoblotting (IB) using anti-FLAG antibody or Coomassie Brilliant Blue (CBB) staining, respectively.

### Immunoblotting

Immunoblotting was performed as described previously [18]. The following antibodies were used: anti-FLAG (1:1,000 dilution), anti-GFP (1:1,000 dilution), anti-β-tubulin (1:1,000 dilution), goat anti-rabbit IgG-HRP (1:2,000 dilution; Santa Cruz Inc.) and goat anti-mouse IgG-HRP (1:2,000 dilution; Santa Cruz Inc.).

### Immunostaining and time-laps imaging analysis

For immunostaining, cells were further cultured before fixing with 4% PFA in PBS or methanol. The fixed cells were rehydrated with 0.3% Triton-X containing PBS, and were incubated at 37°C for 1 h or at 4°C for overnight with primary antibodies as follows: anti-Cep169, anti-CDK5RAP2 (1:100 dilution; sigma), anti-EB1 (1:2,000 dilution; BD Biosciences), anti-FLAG (1:1,000 dilution; WAKO), and anti-β-tubulin (1:1,000 dilution; Sigma-Aldrich). Secondary antibodies (Invitrogen): Alexa Fluor 568 goat anti-mouse IgG (H+L) (1:400; Invitrogen), Alexa Fluor 568 goat anti-rabbit IgG (H+L) (1:400; Invitrogen), Alexa Fluor 488 goat anti-mouse IgG (H+L) (1:400; Invitrogen), and Alexa Fluor goat anti-rabbit 488 (H+L) (1:400; Invitrogen). Immunofluorescence microscopy images were obtained using a Nikon TE2000-E microscope. Live-cell imaging of HeLa expressing EB1-GFP/mRFP-Cep169 were obtained by Olympus FV1000 microscope with a temperature-controlled stage.

#### Statics

Analysis of statistical significance was performed by the Student *t*-test capability in Microsoft Excel.

## Results

### Cep169/NCKAP5L interacts with the CM1 domain of CDK5RAP2

**Fig 1:**
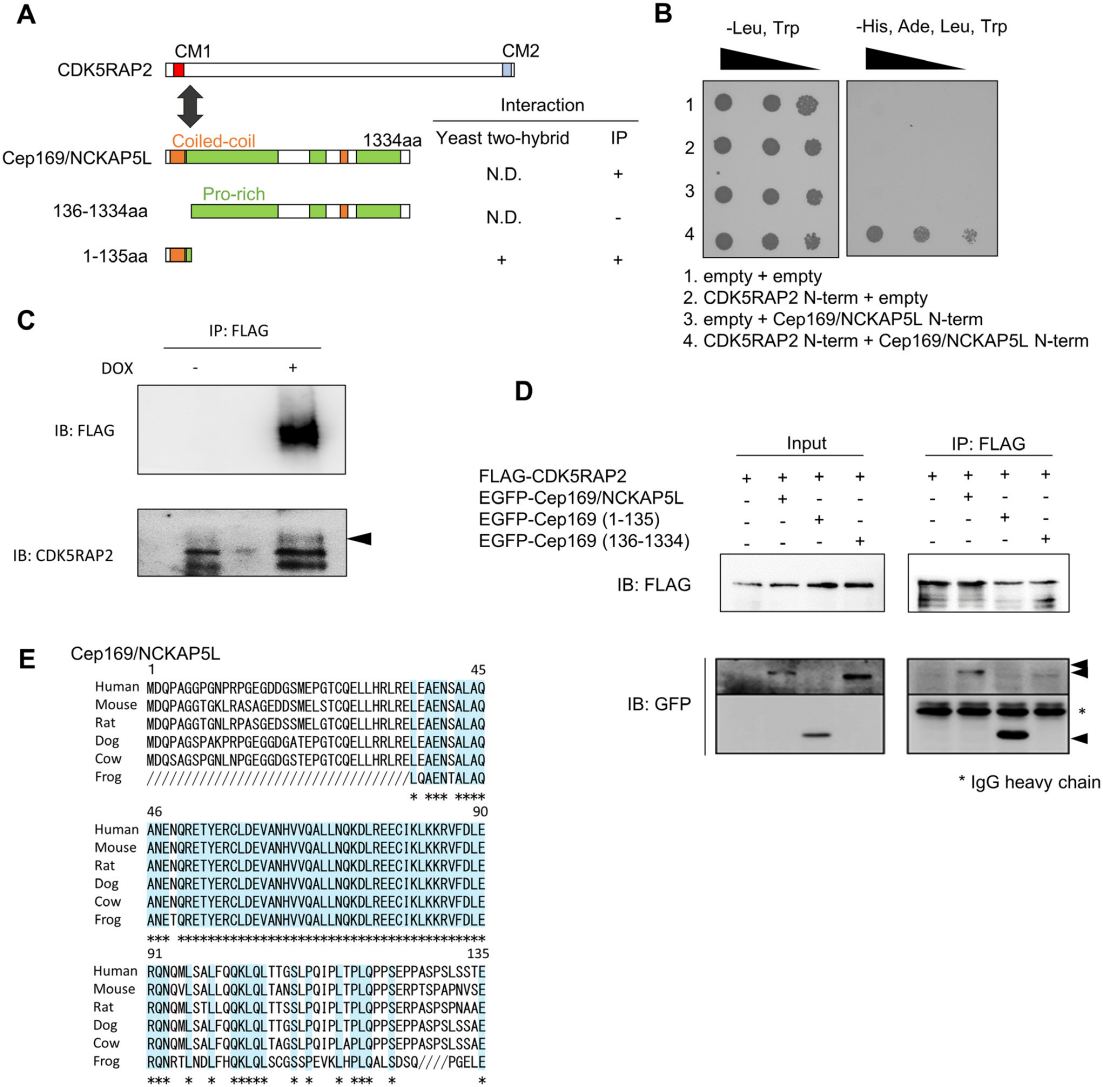
Identification of a CDK5RAP2-interacting protein, Cep169/ NCKAP5L. (**A**) CDK5RAP2, a human homolog of CNN, has two conserved motifs, CM1 (red) and CM2 (blue). The N-terminal region of Cep169/NCKAP5L interacts with the CDK5RAP2 CM1 motif by yeast two-hybrid assay and immunoprecipitation (IP) experiments. The amino acid sequence of the N-terminal region is conserved in vertebrates. (**B**) Interaction of the CM1 motif in CDK5RAP2 with the N-terminal region of Cep169/NCKAP5L, as demonstrated by yeast two-hybrid assay. (**C**) FLAG-Cep169/NCKAP5L interacts with CDK5RAP2 in the doxycycline-inducible FLAG-NCKAP5L HeLa cell line. Complex formation was detected by immunoprecipitation with mouse anti-FLAG antibody, followed by Immunoblotting (IB) with anti-CDK5RAP2 antibody. (**D**) Cep169/NCKAP5L interacts with CDK5RAP2 via its N-terminal region. 293T cells were co-transfected with GFP-Cep169/NCKAP5L wild type or its various deletion mutants together with FLAG-CDK5RAP2. (**E**) Sequence alignment of Cep169/NCKAP5L interaction domain with CDK5RAP2 from different species are as follows: human, mouse, rat, dog, cow, and frog. GenBank accession number of human Cep169/NCKAP5L: NC_000012.11.

To elucidate the function of CDK5RAP2 in centrosomal maturation, we performed a yeast two-hybrid screen using the CM1 of CDK5RAP2 as bait, resulting in the identification of NCKAP5L [19] as an interacting protein (Fig 1A and 1B). Two-hybrid assays revealed that CDK5RAP2 interacts with the amino-terminal region of NCKAP5L (Fig 1B), which has not been characterized functionally until now. To examine whether NCKAP5L interacts with CDK5RAP2 in vivo, we next established doxycycline-inducible FLAG-NCKAP5L HeLa cell lines, and selected the stable one with relatively low expression to avoid the overexpression effect.

Immunoprecipitation assay revealed that the CDK5RAP2 protein was specifically co-immunoprecipitated with FLAG-CDK5RAP2 (Fig 1C). To map the domains involved in the physical interaction between Cep169 and CDK5RAP2, we generated a series of Cep169 deletion mutants (Fig 1D) and performed co-immunoprecipitation assays on cell extracts of 293T cells co-expressing FLAG-CDK5RAP2 and various deletion mutants of GFP-Cep169. GFP-Cep169 full-length and GFP-Cep169 (1–135) (containing residues 1–135), but not GFP-Cep169 (136–1334), was specifically immunoprecipitated with full-length FLAG- CDK5RAP2, indicating that the amino-terminal region of NCKAP5L was necessary and sufficient for its interaction with the CM1 of CDK5RAP2 (Fig 1D).

**Fig 2:**
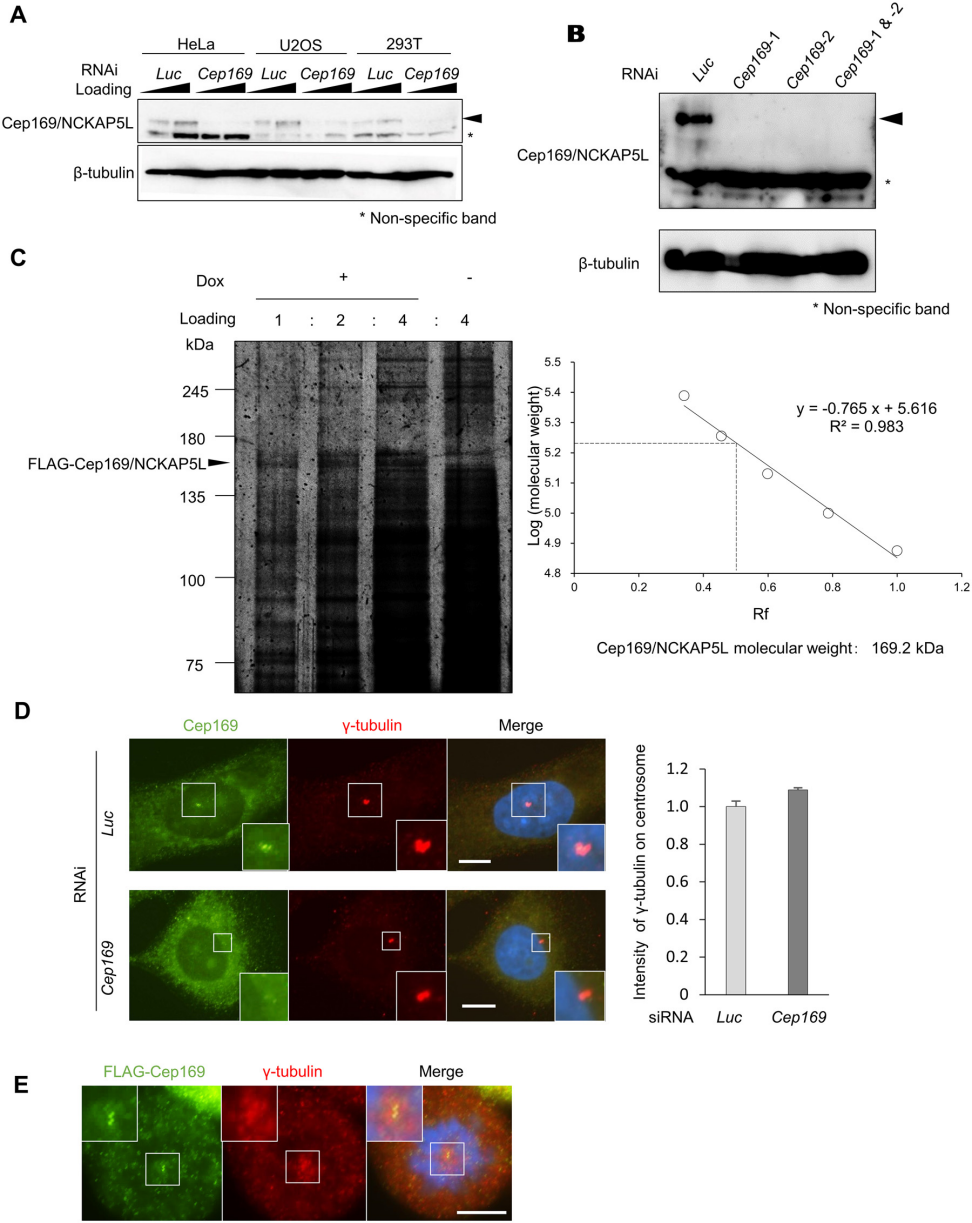
Cep169/NCKAP5L is a centrosomal protein with molecular weight of 169 kDa. (**A**) IB of whole-cell lysates from HeLa, U2OS, and 293T cells treated with control *Luciferase* (*Luc*) siRNA or *Cep169/NCKAP5L* siRNA, and detected with anti-Cep169 and anti-β-tubulin antibodies. (**B**) IB of whole-cell lysates transfected with control (*Luc* siRNA) or two independent siRNA oligonucleotides (*Cep169*-1 and *Cep169*-2 siRNAs), probed with antibodies against Cep169 and β-tubulin. (**C**) Determination of polypeptide molecular size for human Cep169/NCKAP5L by silver staining. The silver staining bands of FLAG-Cep169 observed in the presence of doxycycline disappeared at right lane in the absence of doxycycline. Graph showing estimation of polypeptide size (molecular weight 169 kDa) for Cep169/NCKAP5L. (**D**) Immunofluorescence images of U2OS cells probed with anti-Cep169 and γ-tubulin antibodies (left). (Scale bar, 10 μm.) U2OS cells were treated with control (*Luc* siRNA) or *Cep169/NCKAP5L* siRNA. The centrosomal intensity of *γ*-tubulin in the U2OS cells (right). (*n* = 30 cells for each case; error bars, S.E.). (**E**) Immunofluorescence images of FLAG-Cep169-expressing mitotic HeLa cells probed with anti-FLAG and *γ*-tubulin antibody. (Scale bar, 10 μm.).

**Fig 3:**
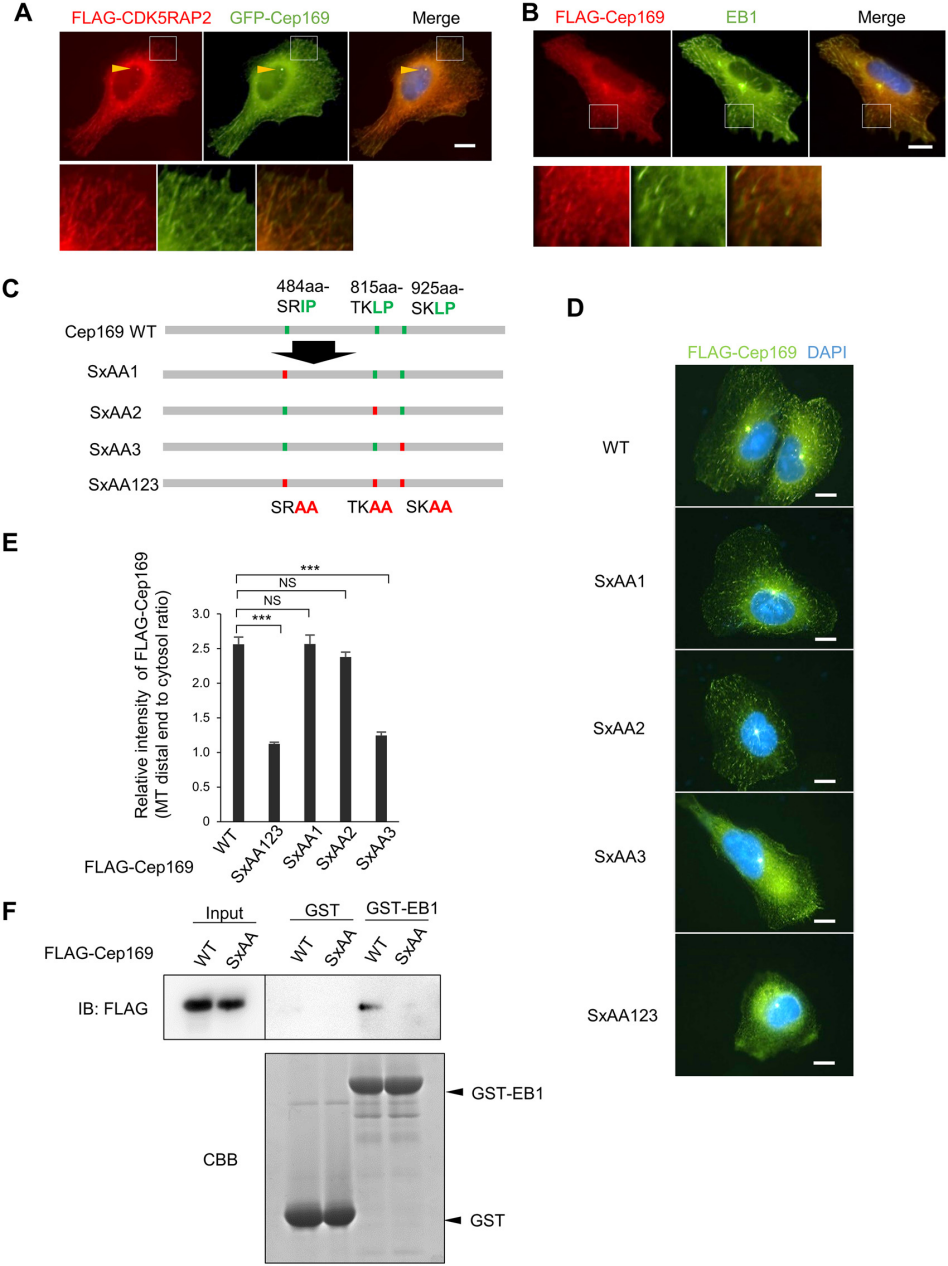
Cep169 targets MT distal ends in a manner dependent on the interaction with EB1. (**A**) Immunofluorescence images showing co-localization of FLAG-tagged CDK5RAP2 and GFP-Cep169 at centrosome (arrowheads) and distal ends in U2OS cells probed with anti-FLAG antibody. (Scale bar, 10 μm). (**B**) Immunofluorescence images showing co-localization of FLAG-Cep169 and EB1 at distal ends in stable U2OS cell probed with anti-FLAG and EB1 antibodies. (Scale bar, 10 μm). (**C**) Schema of Cep169 showing the three (S/T)x(I/L)P motifs (up) and their alanine-substituted mutants (bottom). (**D**) Immunofluorescence image of Cep169 localization in U2OS cells transfected with FLAG-tagged wild type or alanine-substituted mutants of each (S/T)x(I/L)P motif in Cep169, as described in (**C**). (Scale bars, 10 μm). (**E**) Relative intensity of wild type or mutant Cep169 at distal ends. (*n* = 60 cells for each case; error bars, S.E.). *P* values result from *t*-test (***P < 0.0001, NS: not significant). (**F**) Pull-down assays using recombinant GST-EB1 as an affinity matrix to isolate FLAG-Cep169 WT or SxAA123 mutant. FLAG-Cep169 or GST and GST-EB1 proteins were analyzed with IB using anti-FLAG antibody or Coomassie Brilliant Blue (CBB) staining, respectively.

In a search of GenBank databases, we identified putative NCKAP5L sequences from several vertebrate species, including mouse, rat, dog, cow, and frog; analysis of these sequences revealed that the CDK5RAP2 interaction is mediated through a highly conserved segment in the NCKAP5L amino-terminal region (Fig 1E), suggesting that its interaction with CDK5RAP2 is conserved during evolution. Human NCKAP5L protein contains two coiled-coil domains and three proline-rich domains. To characterize NCKAP5L, we generated a polyclonal rabbit antibody against its recombinant amino-terminal region, because the function of these homologues is not reported at all until now. Immunoblotting of HeLa cell lysates with the affinity-purified antibody revealed specific endogenous NCKAP5L bands in HeLa, U2OS, and 293T cells (Fig 2A and 2B). To confirm that the 169 kDa band was due to NCKAP5L, we disrupted the expression of endogenous NCKAP5L by RNAi. As shown in Fig 2A and 2B, the level of endogenous NCKAP5L was significantly reduced relative to controls by two independent short interfering RNA (siRNA) oligonucleotides (*Cep169*-1 and *Cep169*-2 siRNAs). To determine the accurate molecular weight, FLAG-NCKAP5L was immunoprecipitated with FLAG antibody from extracts of the HeLa stable cell line treated with doxycycline. The molecular weight of the FLAG-NCKAP5L was consistent with 169 kDa by silver staining on SDS-PAGE (Fig 2C). The antibody specifically recognized a centrosomal protein with the expected molecular weight of 169 kDa, in immunoblotting, we renamed NCKAP5L as Cep169 (centrosomal protein of 169 kDa). Immunostaining of U2OS or HeLa cells using the polyclonal antibody revealed that Cep169 localized adjacent to γ-tubulin, a marker of centrosome, in both interphase and mitosis (Fig 2D and 2E). In order to confirm whether the centrosomal localization detected by the Cep169 antibody is due to Cep169 protein, we observed the effect of Cep169 depletion on the centrosomal staining. In the Cep169 RNAi cells, the centrosome staining was significantly reduced (Fig 2D). In addition, GFP-Cep169 also colocalized with FLAG-CDK5RAP2 at centrosomes (Fig 3A). Interestingly, depletion of Cep169 didn’t diminish γ-tubulin intensity at centrosomes (Fig 2D). These data suggest that Cep169 is not required for assembly of γ-tubulin onto centrosomes. Next, we investigated whether centrosomal localization of both Cep169 and CDK5RAP2 require each other. Depletion of Cep169 by RNAi has no effect on the centrosomal localization of FLAG-tagged or endogenous CDK5RAP2 in the U2OS cells (S1 Fig). Similarly, depletion of CDK5RAP2 has no effect on the centrosomal localization of FLAG-tagged or endogenous Cep169 (S1 Fig). Thus, Cep169 and CDK5RAP2 are not mutually interdependent for their localization to centrosomes.

### Cep169 tracks growing MT tips through SxIP EB1-binding motifs

The ends of growing MTs accumulate +TIPs, a diverse set of factors that control MT dynamics and organization. One of these factors, EB1, forms the core machinery for MT tip tracking and targets additional +TIPs to MT ends. CDK5RAP2 interacts directly with EB1 at the MT plus-end through a short hydrophobic (S/T)x(I/L)P sequence motif (SxIP) (Fig 3A and 3B) [16]. Similarly, Cep169 also contains three SxIP motifs, which are highly conserved (Fig 3C). In U2OS cells, both FLAG-Cep169 and EB1 signals formed filamentous structures co-aligned with MTs (Fig 3B). These patterns were most apparent near the extending edge of cell protrusions, where FLAG-CDK5RAP2 labeling was concentrated at MT distal tips (Fig 3A). The filamentous patterns detected by anti-Cep169 antibody were eliminated upon Cep169 depletion, indicating that they were specific to Cep169 (data not shown).

If Cep169 is a member of the +TIPs, it should track the growing MT plus-ends. Time-lapse microscopy revealed that GFP- or mRFP-Cep169 exhibited typical +TIP motion in U2OS cells (Fig 3A and S1 Movie). We next asked whether Cep169 association with MT plus-ends requires EB1-binding SxIP motifs. To address this question, we transiently transfected U2OS cells with FLAG-Cep169 or the EB1-binding-deficient mutants SxAA1, SxAA2, SxAA3, and SxAA123 (Fig 3D and 3E). Two of these SxIP motif mutants (SxAA3 and SxAA123) significantly decreased the percentage of cells in which FLAG signal localized to the majority of observed EB1 comets at the growing MT plus-ends. Quantitation revealed that among the three SxIP motifs, the C-terminal SKLP is primarily responsible for localization of Cep169 at MT distal tips. We further performed pull-down assays using recombinant GST-EB1 as an affinity matrix to isolate FLAG-Cep169 WT or SxAA123 mutant from 293T cell extracts. As shown in Fig 3F, FLAG-Cep169 WT, but not SxAA123, bound GST-EB1. These results strongly suggest that Cep169 tracks growing MT TIP through its SxIP motifs in an EB1-dependent manner.

### Cep169 regulates MT stability

**Fig 4:**
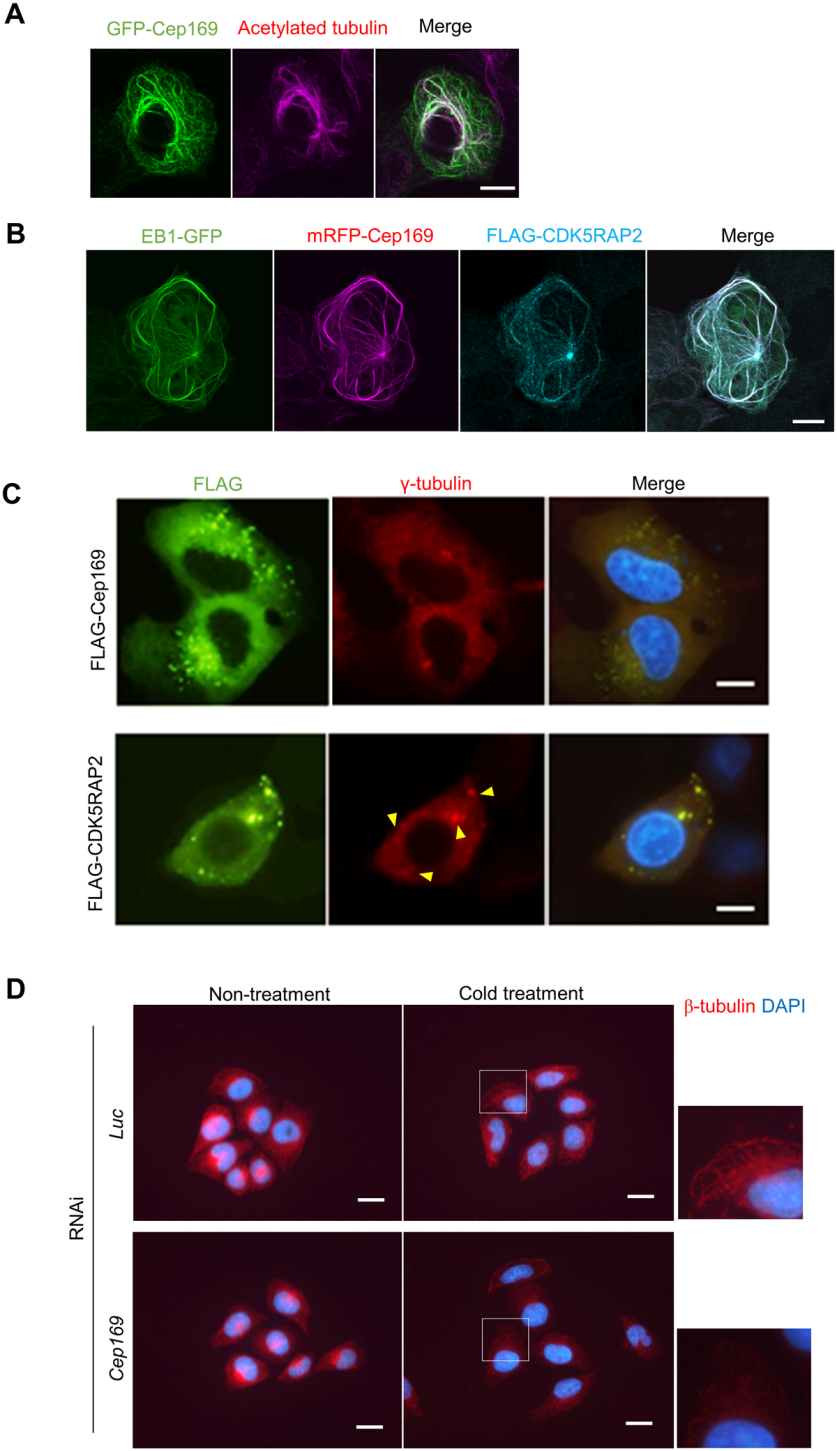
Cep169 regulates MT dynamics. (**A**) U2OS cells were transiently transfected with GFP-Cep169. The cells were stained for acetylated MTs (anti-acetylated α-tubulin antibody). (Scale bar, 10 μm). (**B**) U2OS cells stably expressing EB1-GFP were transiently transfected with mRFP-Cep169 and FLAG-CDK5RAP2. The cells were stained for anti-FLAG antibody. (Scale bar, 10 μm). (**C**) CDK5RAP2, but not Cep169, has MT organizing center (MTOC) activity. U2OS cells were transfected with FLAG-CDK5RAP2 or FLAG-Cep169 and immunostained with anti-FLAG and anti-*γ*-tubulin antibodies. Expression of CDK5RAP2, but not Cep169, caused formation of cytoplasmic aggregates (arrowheads) associated with *γ*-tubulin. (Scale bars, 10 μm). (**D**) Immunofluorescence images of cells probed with anti-β-tubulin antibody (red) and DAPI (blue) in control *Luc* siRNA cells or *Cep169* siRNA cells and then fixed after cold treatment for 15 min. (Scale bars, 20 μm).

CDK5RAP2-EB1 complex induces MTs bundling and acetylation, indicating that CDK5RAP2-EB1 regulates MT dynamics and stability [16]. Thus, we next investigated the role of Cep169 in the MT regulation and observed the effect of Cep169 expression on MT organization in U2OS cells by transient expression. Interphase MTs of control U2OS cells developed fine filamentous networks, which radiated from MT organizing centers (data not shown). Expression of GFP-Cep169 into U2OS cells induced the formation of long MT bundles emanating from the centrosomes (Fig 4A). Remarkably, a subset of interphase cells expressing GFP-Cep169 showed bright cytoplasmic fibers that colocalized with aberrantly thickened, curly and intertwined MTs, reminiscent of the MT bundle formation induced by expression of CDK5RAP2 [16]. Moreover, Cep169 expression induced intense acetylation of MTs, pointing to MT stabilization (Fig 4A). Intriguingly, in these cells, Cep169, CDK5RAP2 and EB1 were enriched on the same bundles (Fig 4B).

Because CM1 of CDK5RAP2 is required for recruitment of γ-TuRC onto centrosomes, loss of CDK5RAP2 delocalizes centrosomal γ-tubulin and disorganizes MTs [5]. We next investigated the role of Cep169 in the MT-organizing function of centrosomes. Depletion of CDK5RAP2, but not Cep169, significantly diminished γ-tubulin at centrosomes in interphase cells. Overexpression of CDK5RAP2, but not Cep169, induced formation of cytoplasmic foci that can recruit γ-tubulin (Fig 4C). This is consistent with our observation showing that depletion of Cep169 has no effect on the assembly of γ-tubulin onto centrosomes in interphase cells (Fig 2D). These data suggest that Cep169 is dispensable for recruitment of γ-tubulin onto centrosomes and plays no essential role in the MT-organizing center and centrosomal maturation.

We next investigated the effect of Cep169 on MT stabilization by cold-induced MT depolymerization assay. We depolymerized MTs with cold-treatment for 15 min and then fixed. The MT depolymerization rate seems to be significantly elevated in Cep169-depleted cells during interphase compared with control cells (Fig 4D). These data strongly suggest that Cep169 is required for stability of MTs, although Cep169 is dispensable for the assembly of γ-tubulin onto centrosomes and centrosomal maturation process in CDK5RAP2 function.

## Discussion

CDK5RAP2 has multiple functions in the control of MT organization. These functions include recruitment of γ-tubulin onto centrosomes, mitotic spindle position, and MT plus-end regulation [5, 16, 20]. CDK5RAP2 and its related *Drosophila* protein CNN share homology in two regions, CM1 and CM2, located at the N and C termini, respectively [3, 21]. CM1 of CDK5RAP2 is important for assembly of γ-tubulin onto centrosomes. In present studies, we showed identification of a novel MT plus-end-tracking and centrosomal protein, Cep169, as a CDK5RAP2-interacting protein and characterization of its functions. Cep169 directly interacts with CM1 of CDK5RAP2. The amino-terminal domain of Cep169 required for interaction with CDK5RAP2 is highly conserved between human and frog, which suggest the possibility of conserved function of Cep169 with CDK5RAP2 during evolution (Fig 1E). In cells, Cep169 associates with interphase centrosomes and mitotic spindle poles. In addition, Cep169 associates with MT distal ends and plays a role in MT dynamics. Both Cep169 and CDK5RAP2 belong to a class of +TIPs that contains the EB1-binding a short hydrophobic (S/T)x(I/L)P sequence motif (SxIP). +TIPs are specifically and directly concentrated at the growing plus-ends of MTs with EB1, which physically interacts with an array of +TIPs to recruit them to MT plus-ends [8, 22]. The dipeptide Leu938-Pro939 of SxIP motif within basic and Ser-rich sequence of CDK5RAP2 plays crucial role in EB1 interaction [16]. We have also found that Ala substitution for Leu927-Pro928 (SxAA3) ablates the EB1 interaction with Cep169, although it has three putative SxIP motifs. In the cells, almost all Cep169 protein with SxAA3 disappears from all growing MT ends, indicating the C-terminal SKLP motif of Cep169 is responsible for interaction with EB1. The EB1-interaction motif is highly conserved in the sequences between human, mouse, rat, dog, cow, and frog. These data indicate that +TIPs function may be conserved in the Cep169 family.

CDK5RAP2 functions in both centrosomal recruitment of γ-TuRC and regulation of MT dynamics and stability. However, Cep169 is not required for recruitment of γ-tubulin onto centrosomes (Figs 2D and 4C), and depletion of Cep169 had no effect on MT nucleation by MT regrowth assay in mitotic HeLa cells (data not shown). These data suggest that Cep169 might possess other function of CDK5RAP2, although it directly interacts with CM1 of CDK5RAP2, which is required for recruitment of γ-TuRC onto centrosomes. It was reported that the mere stabilization of MTs regulates MTs reorganization and dynamics, because drugs which stabilize MTs, such as taxol, can induce this altered organization in fibroblasts [23]. We found that Cep169 promotes MT growth and MT bundling formation, and it stabilizes MTs by increasing intense acetylation of MTs (Fig 4A). The observed MT-bundling and -stabilizing activities of Cep169 are similar to those of CDK5RAP2 [16]. This result is consistent with the recent report, which showed that CDK5RAP2-EB1 complex induces MTs bundling and acetylation, indicating that CDK5RAP2-EB1 regulates MT dynamics [16]. Indeed, Cep169 interacts with CDK5RAP2 through its N-terminal domain, and N-terminal deletion mutant of Cep169 has no MT-bundling and –stabilizing activities. These results suggest the possibility that Cep169 regulates MT organization and stabilization in concert with CDK5RAP2. However, we cannot exclude the possibility that the bundling activity of Cep169 is partially due to the overexpression of Cep169. Thus, physiological relevance of the bundling activity needs to be addressed. Interestingly, MT depolymerization assay showed that Cep169 is required for MT stabilization. Although CDK5RAP2 is also required for MT stability, whether Cep169 and CDK5RAP2 work in collaboration for regulation of MT dynamics as +TIPs and MT stabilization is currently under investigation.

It is well known that MT-associated proteins (MAPs) play an essential role in nerve cell processes. Expression of Tau or MAP2A in fibroblasts reorganizes interphase MTs into MT bundles, and that the depletion of Tau or MAP2A results in the inhibition of axon or neurite elongation, respectively [24, 25]. Autosomal Recessive Primary Microcephaly (MCPH) is characterized by small brain size due to deficient neuron production in the developing cerebral cortex. Intriguingly, CDK5RAP2 is highly expressed in the neural progenitor pool and its mutations cause MCPH [26]. If CDK5RAP2-mediated MT organization is linked to mechanism that underlies the development of MCPH, Cep169 may involve in neurogenesis program with CDK5RAP2.

CNN family is highly conserved between yeast and human. In contrast, Cep169 has potential homologues in only vertebrates but not in lower eukaryotes such as yeast, worm, and fly. Thus, we speculate that Cep169 emerges at a later stage in evolution, and modulates additional function of CDK5RAP2 as a higher-eukaryote-specific partner.

## Supporting Information

S1 Fig Cep169 and CDK5RAP2 are not mutually interdependent for their localization to centrosomes and MT plus-ends. Cep169 is not required for localization of CDK5RAP2 at the centrosomes and MT distal ends. Immunolocalization of *γ*-tubulin and FLAG-CDK5RAP2 (**A**) or endogenous CDK5RAP2 (**B**) at centrosomes in U2OS cells transfected with control *Luc* or *Cep169* siRNAs. (Scale bars, 10 μm). The centrosomal intensity of Cep169 in the U2OS cells (*n* = 30 cells for each case; error bars, S.E.). CDK5RAP2 is not required for localization of Cep169 at the centrosomes and MT distal ends. Immunolocalization of FLAG-Cep169 (**C**) or endogenous Cep169 (**D**) at centrosomes in U2OS cells transfected with control *Luc* or *CDK5RAP2* siRNAs. (Scale bars, 10 μm). The centrosomal intensity of CDK5RAP2 in the U2OS cells (*n* = 30 cells for each case; error bars, S.E.).
(TIF)

S1 Movie HeLa cells expressing EB1-GFP and mRFP-Cep169 were filmed through interphase. Cep169 exhibited highly dynamic comet-like fluorescence patterns along the growing MT tips and moved toward the cell periphery. Images were taken by confocal microscopy every 3 sec.
(MOV)
