# Supplementary figures and images for: Cep169, a Novel Microtubule Plus-End-Tracking Centrosomal Protein, Binds to CDK5RAP2 and Regulates Microtubule Stability
Source: PLoS One. 2015 Oct 20;10(10):e0140968. doi: 10.1371/journal.pone.0140968 (PMC4613824; doi:10.1371/journal.pone.0140968)

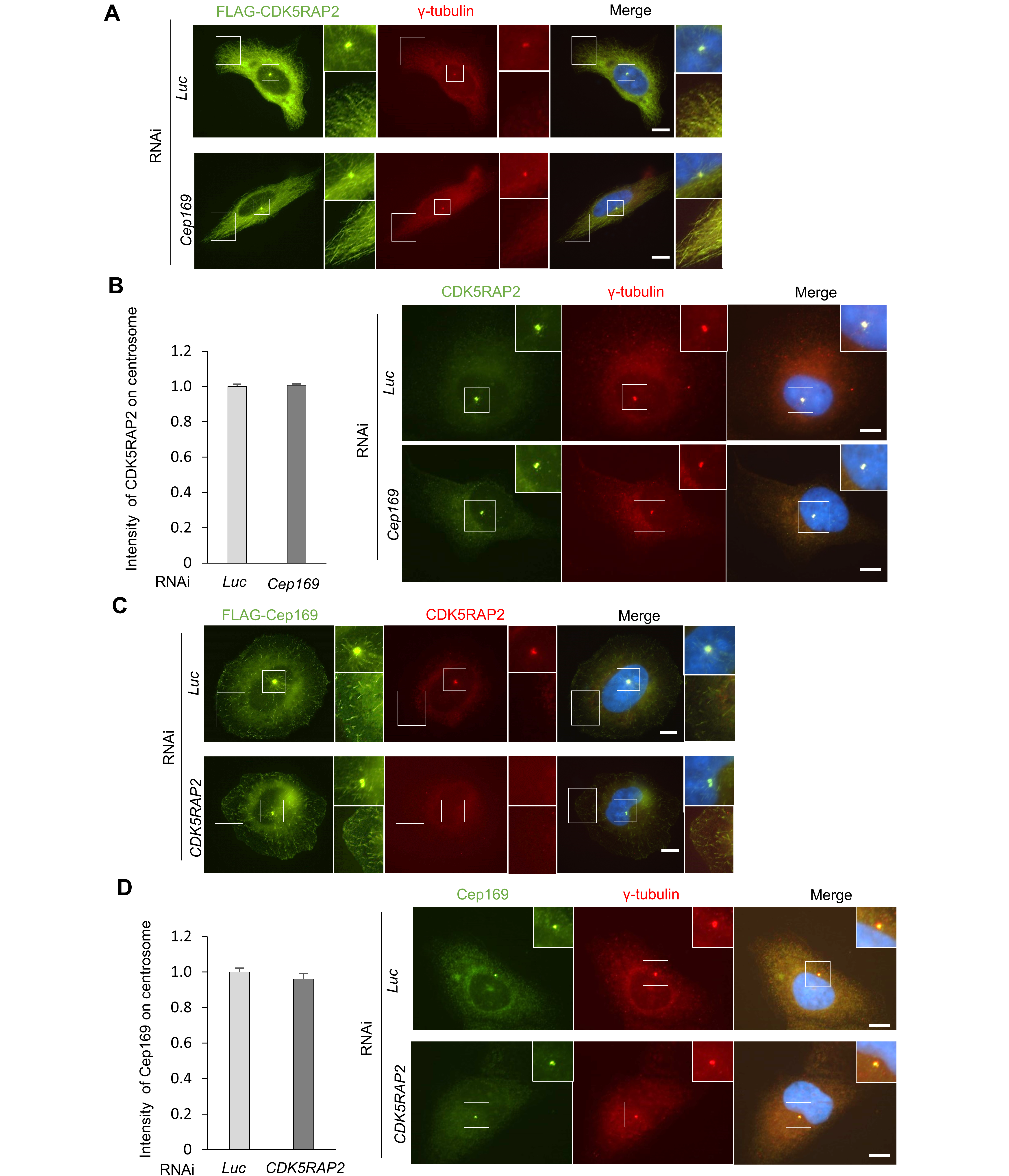

Supplement: S1 Fig — Cep169 is not required for localization of CDK5RAP2 at the centrosomes and MT distal ends. Immunolocalization of γ-tubulin and FLAG-CDK5RAP2 (A) or endogenous CDK5RAP2 (B) at centrosomes in U2OS cells transfected with control Luc or Cep169 siRNAs. (Scale bars, 10 μm). The centrosomal intensity of Cep169 in the U2OS cells (n = 30 cells for each case; error bars, S.E.). CDK5RAP2 is not required for localization of Cep169 at the centrosomes and MT distal ends. Immunolocalization of FLAG-Cep169 (C) or endogenous Cep169 (D) at centrosomes in U2OS cells transfected with control Luc or CDK5RAP2 siRNAs. (Scale bars, 10 μm). The centrosomal intensity of CDK5RAP2 in the U2OS cells (n = 30 cells for each case; error bars, S.E.). (TIF) [file pone.0140968.s001.tif]
